# The discovery BPD (D-BPD) program: study protocol of a prospective translational multicenter collaborative study to investigate determinants of chronic lung disease in very low birth weight infants

**DOI:** 10.1186/s12887-019-1610-8

**Published:** 2019-07-06

**Authors:** Gaston Ofman, Mauricio T. Caballero, Damian Alvarez Paggi, Jacqui Marzec, Florencia Nowogrodzki, Hye-Youn Cho, Mariana Sorgetti, Guillermo Colantonio, Alejandra Bianchi, Luis M. Prudent, Nestor Vain, Gonzalo Mariani, Jorge Digregorio, Elba Lopez Turconi, Cristina Osio, Fernanda Galletti, Mariangeles Quiros, Andrea Brum, Santiago Lopez Garcia, Silvia Garcia, Douglas Bell, Marcus H. Jones, Trent E. Tipple, Steven R. Kleeberger, Fernando P. Polack

**Affiliations:** 1 Department of Pediatrics, University of Alabama at Birmingham, Birmingham, USA; 2 Fundación INFANT, Buenos Aires, Argentina; 3 National Scientific and Technical Research Council of Argentina, Buenos Aires, Argentina; 4 National Institute of Environmental Health Sciences, North Carolina, USA; 5 Fundación para la salud materno infantil, Buenos Aires, Argentina; 6 Universidade Católica do Rio Grande do Sul, Porto Alegre, Brazil; 7 Clinica y Maternidad Suizo Argentina, Buenos Aires, Argentina; 8 Sanatorio de la Trinidad, Buenos Aires, Argentina; 9 Hospital Italiano de Buenos Aires, Buenos Aires, Argentina; 10 Sanatorio de los Arcos, Buenos Aires, Argentina; 11 Sanatorio Otamendi y Miroli, Buenos Aires, Argentina

## Abstract

**Background:**

Premature birth is a growing and serious public health problem affecting more than one of every ten infants worldwide. Bronchopulmonary dysplasia (BPD) is the most common neonatal morbidity associated with prematurity and infants with BPD suffer from increased incidence of respiratory infections, asthma, other forms of chronic lung illness, and death (Day and Ryan, Pediatr Res 81: 210–213, 2017; Isayama et la., JAMA Pediatr 171:271–279, 2017). BPD is now understood as a longitudinal disease process influenced by the intrauterine environment during gestation and modulated by gene-environment interactions throughout the neonatal and early childhood periods. Despite of this concept, there remains a paucity of multidisciplinary team-based approaches dedicated to the comprehensive study of this complex disease.

**Methods:**

The Discovery BPD (D-BPD) Program involves a cohort of infants < 1,250 g at birth prospectively followed until 6 years of age. The program integrates analysis of detailed clinical data by machine learning, genetic susceptibility and molecular translation studies.

**Discussion:**

The current gap in understanding BPD as a complex multi-trait spectrum of different disease endotypes will be addressed by a bedside-to-bench and bench-to-bedside approach in the D-BPD program. The D-BPD will provide enhanced understanding of mechanisms, evolution and consequences of lung diseases in preterm infants. The D-BPD program represents a unique opportunity to combine the expertise of biologists, neonatologists, pulmonologists, geneticists and biostatisticians to examine the disease process from multiple perspectives with a singular goal of improving outcomes of premature infants.

**Trial registration:**

Does not apply for this study.

**Electronic supplementary material:**

The online version of this article (10.1186/s12887-019-1610-8) contains supplementary material, which is available to authorized users.

## Background

Premature birth is a serious public health problem affecting more than one of every 10 infants worldwide [1]. Bronchopulmonary dysplasia (BPD), defined by a requirement for oxygen supplementation at 36 weeks post-conceptional age (PCA) due to respiratory insufficiency. BPD is the most common neonatal morbidity and is associated with increased incidence of infections, asthma, other forms of chronic lung illness, and death [2, 3]. Very low birth weight (VLBW) infants (BW < 1,250 g) are at greatest risk of developing BPD and disproportionately experience long-term consequences of prematurity [4, 5].

While VLBW infants often require treatment for pulmonary complications after birth, their course upon graduation from the neonatal intensive care unit (NICU) is highly variable. Results from the NHLBI Prematurity and Respiratory Outcomes Program (PROP) revealed that some though some infants remain asymptomatic and appear to live a healthy first year of life despite of a diagnosis of BPD, others experience frequent hospitalizations for respiratory indications, need for home respiratory support and suffer from additional respiratory morbidities [6–9]. Long term, a significant proportion of former VLBW infants, with or without BPD, exhibit respiratory limitations at school age and into adulthood [10–12]. Predicting the long-term pulmonary outcomes for VLBW infants early in life is difficult, despite ~ 30% of infants receiving a diagnosis of BPD during their initial hospitalization. This challenge is due, in part, to the definition of BPD itself. While a diagnosis of BPD simply identifies babies requiring oxygen therapy relatively early after birth, limited information is available during the first months of life to predict the evolution of lung growth and development and the impact on gas exchange. BPD likely represents a diagnostic umbrella encompassing a broad range of pulmonary diseases of diverse etiologies and prognoses (endotypes). This hypothesis is supported by the absence of genetic studies that identify single genes that strongly correlate with BPD and conclusively predict long term respiratory compromise in prematurely born infants [13, 14].

Environmental exposures of the developing lung are recognized as a key factors that influence long-term outcomes [15] and modulation of these exposures may offer a window of opportunity to improve the undesirable consequences of lung immaturity. In addition, understanding patterns of lung disease within the BPD umbrella – particularly when using an unbiased approach like machine learning [16] - may enable redefinition of lung diseases in VLBW infants with greater linkage between phenotype, genetic, and/or environmental determinants of disease. Given the gaps in our understanding of lung disease endotypes in prematurely born infants, the molecular bases underlying these endotypes, the genetic predisposition toward individual endotypes, and the contribution(s) of environmental factors in disease inception and severity, we established the Discovery BPD program (D-BPD). D-BPD is a multi-disciplinary, seven center program (Table 1) that fosters collaboration between neonatologists, pulmonologists, immunologists, environmental biologists, basic scientists and bioinformaticians. The D-BPD collaborative will enable identification of new endotypes within the BPD umbrella and define genetic, molecular and environmental factors associated with pathogenesis.

**Table 1:** Participating centers and specific projects

Program center	Center objective	Enrollment
National Institute of Environmental Health Sciences (NIEHS)	Biorepository analysis for D-BPD	N/A
Fundacion INFANT	Data coordinating center for the D-BPD Molecular translation of genetic mutations	N/A
University of Alabama at Birmingham (UAB)	Laboratory research/Redox biology	N/A
Pontificia Universidade Católica Laboratory of Respiratory Physiology	FOT coordination and analysis	N/A
Clinica y Matenidad Suizo Argentina	Recruitment	104
Sanatorio Otamendi y Miroli	Recruitment	47
Sanatorio de la Trinidad	Recruitment	47
Sanatorio de los Arcos	Recruitment	52
Hospital Italiano de Buenos Aires	Recruitment	75

**Fig. 1:**
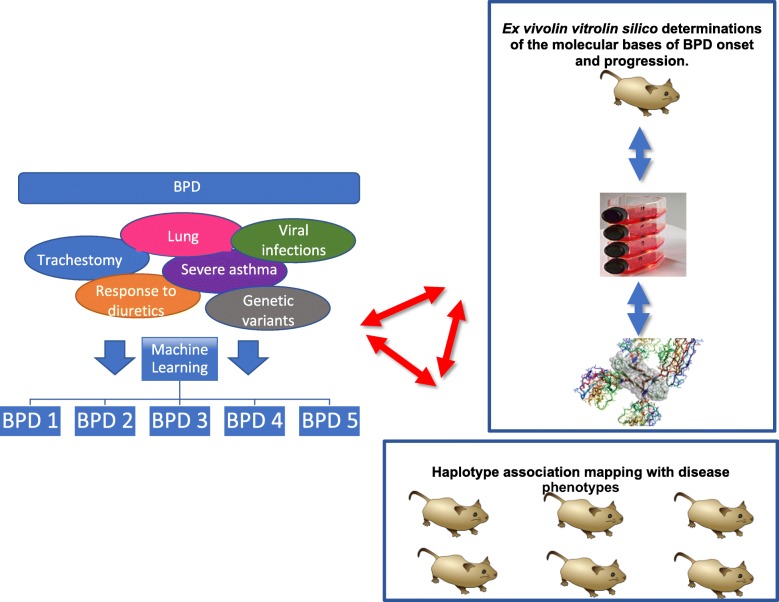
The D-BPD research areas integration. The clinical data core, using machine learning strategies will leverage the detailed longitudinal clinical data the gene susceptibility program using genome-wide association mapping and positional cloning in murine strains to identify candidate susceptibility genes, and a basic science molecular program exploring mechanistic correlates of clinical and genetic findings associated with BPD endotypes. Image credits: Wikimedia Commons

D-BPD integrates three distinct yet interactive areas of research (Fig. 1). The clinical data core uses machine learning strategies to leverage the detailed longitudinal clinical data. The gene susceptibility program uses genome-wide association mapping and positional cloning in inbred strains of mice to identify candidate susceptibility genes. Finally, the basic science molecular program explores the mechanistic correlates of clinical and genetic findings associated with oxidative stress. A list of all investigators and research staff from each center is provided in Additional file 1.

As of this writing, the D-BPD cohort currently includes 325 infant/mother/father triads. Infants < 1,250 g at birth will be followed until 6 years of age. In this manuscript, we present the D-BPD program protocol, illustrate the breadth of data and biospecimens available for study, and outline ongoing and future investigations that will enable the identification of preventive strategies against lung diseases of prematurity.

## Methods

**Fig. 2:**
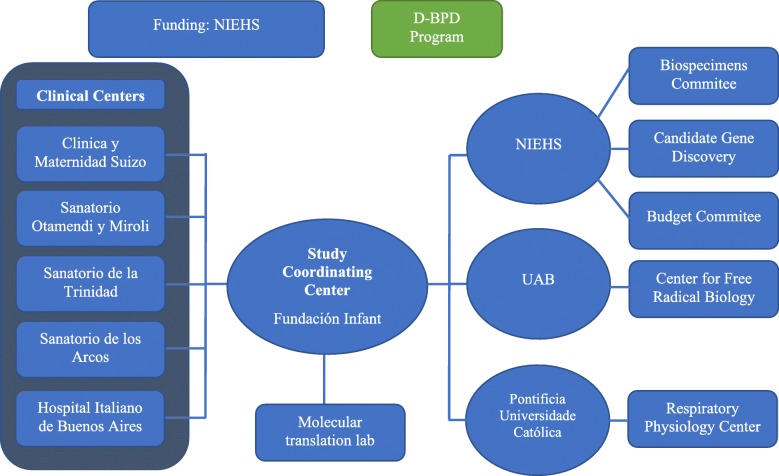
Discovery BPD (D-BPD) structure

The D-BPD structure is depicted in Fig. 2. Five clinical centers are coordinated by Fundacion INFANT through the Preterm INFANT Network. Fundacion INFANT is responsible for supervising the conduct of the clinical study, including data collection, regulatory affairs, and sample collection, early processing and storage. Fundacion INFANT and the National Institute of Environmental Health Sciences (NIEHS) monitor quality collection of data through clinical report forms. Oversight of the program rests in an NIEHS appointed Steering Committee Chair, NIH officials, and an Observational and Safety Monitoring Board (OSMB). Teams from the NIEHS, Fundación INFANT, the University of Alabama at Birmingham (UAB) and the Pontificia Universidade Católica do Rio Grande do Sul conduct every other week videoconference calls to discuss all aspects of the program including recruitment, data collection, new data, recent results, long term objectives, and regulatory matters.

### Multicenter protocol development

#### Outcomes of interest

The primary aim of D-BPD is to identify new endotypes within the BPD umbrella in order to define genetic, molecular and environmental factors associated with disease pathogeneses. These data will enable the prediction of respiratory morbidity through early childhood. Long-term lung disease determinations in D-BPD will be assessed by the combined clinical evaluation of respiratory signs and symptoms until the age of 6 years using physiologic evaluations of lung function at defined time points during childhood. The D-BPD program will also define genetic, molecular and environmental factors associated with the traditional definition of BPD, its severity, and the inception and evolution of other prematurity morbidities and death.

### Protocol

**Fig. 3:**
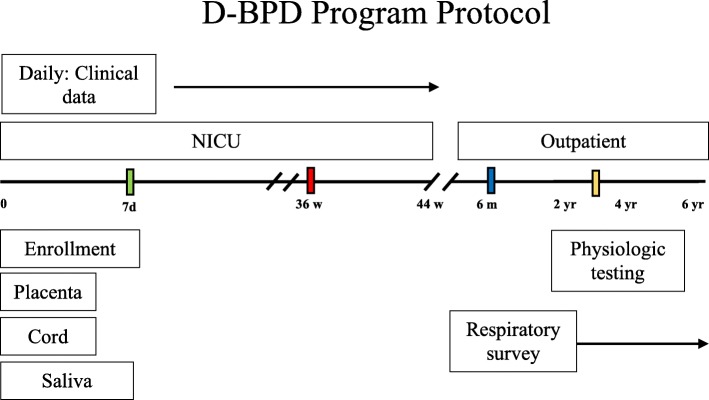
D-BPD Program Protocol Time Line spans from birth to 6 years of corrected age collecting health data and biospecimens. The babies will be monitored daily during their NICU stay by the participating neonatologist (without direct clinical responsibilities) using structured data collection log sheets. Information of the clinical course will be collected daily during the first 28 days and every 2 days thereafter. Afterwards phone calls will be made every 6 months until 6 years of age are completed

The inclusion and exclusion criteria are listed in Table 2. The protocol is outlined in Fig. 3. The protocol integrates data from the molecular to population-level. We expect to enroll 750 infants. Based on prior population studies, we estimate that 40% of this cohort will meet the diagnoses of BPD. With these parameters, the study has more than 80% power to compare an area under the curve (AUC) larger than 0.6 in a receiver operating characteristic (ROC) analysis, against a null hypothesis of an AUC with no diagnostic value (AUC = 0.5). This is a conservative estimate, as the power is larger for AUC values larger than 0.6.

**Table 2:** Inclusion/Exclusion criteria

Inclusion criteria	
- Birth weight < 1250 g	
Exclusion criteria	
- Structurally significant heart disease	
- Congenital anomalies of the respiratory tract	
- Eye malformations	
- Immunodeficiencies	
- Conception by in vitro fertilization

### Environmental and clinical data collection

Parents who consent to participate in the study are personally interviewed by participating neonatologists using questionnaires specifically designed by the NIEHS epidemiologists and biostatisticians for this study. This questionnaire collects epidemiological and clinical information associated with pregnancy. Data from VLBW infants are obtained prospectively every day during the NICU stay using specially designed forms. After discharge, families are contacted via telephone and interviewed using modified ISAAC questionnaires to monitor the respiratory status of their baby. These questionnaires have been modified to assess respiratory health at 6 months and yearly thereafter up to 6 years PCA.

### Biospecimen archive (bedside to bench)

The characterization of long-term respiratory outcomes in VLBW infants is hindered by the absence of biological materials to study phenotype-specific disease determinants, from molecular alterations in mitochondrial function to genetic mutations or gene-by-environment interactions. NIEHS and Fundación INFANT, in conjunction with the Preterm Network, established standardized procedures for sample collection and central processing, and protocols for accessing the resulting biorepositories. Saliva specimens from parents are collected at study entry for DNA extraction. Infant saliva samples are obtained in the first 4 weeks of life. Early (birth) specimens allow for exploration of injuries and exposures during gestation, developmental and genetic biosynthetic capacities. Collection at later time points (after 1 week) likely reflect responses to oxidative stress, infection, inflammation, nutritional state, and tissue repair. The program is now collecting samples from placenta tissue and umbilical cord blood tissue at the time of birth.

### Assessments of respiratory function (physiologic biomarkers)

The evaluation of lung function in early years of life has been hampered by the need for sedation. In addition, the absence of appropriate biomarkers for the inception of asthma contributed to the scarcity of tools to predict long-term lung health in infancy. Forced oscillatory test (FOT) uses the patient’s spontaneous respiration without sedation to define the physiology of the small and large airways. FOT applies an oscillation pressure wave generated by a loudspeaker to the respiratory system to analyze the pressure-flow relationship in terms of impedance [Zrs; encompasses both resistance (Rrs) and reactance (Xrs)]. Rrs, calculated from pressure and flow signals, is a measure of central and peripheral airway caliber, while Xrs, derived from the pressure in the phase with volume, relates to compliance (Crs) and inertance (Irs). FOT has been used to detect lung function abnormalities in asthmatics with normal spirometry [17], to identify the deleterious effects of oxidative stress (e.g., cigarette smoke exposure) on pulmonary function, and to study bronchodilator responsiveness in infants [18]. Therefore, we will use FOT to evaluate lung function in study participants between the age of 3–4 years. Participants will again be evaluated at the age of 5–7 years.

### Data collection, management and storage systems

All source documents and laboratory reports are reviewed by the clinical team and the staff in charge of data entry to ensure that they are accurate and complete. Data collection is performed by clinical trial staff at the sites under supervision of the PI. During the study, investigators maintain complete and accurate documentation. Research sites that participate in this study maintain maximum confidentiality about the clinical and research information obtained from study participants. All information about study participants is kept in password-protected computer files or in locked cabinets accessible only to authorized personnel. Biological samples, tables, and files are identified by unique numbers. Questionnaire data are entered twice in the database designed by NIEHS for such purpose. This database is reviewed and maintained by the data manager.

### Genetic susceptibility

In order to explore the phenotypic variation attributable to gene-environment interaction, the NIEHS has designed a process to translate findings in model organisms to human disease susceptibility in order to draw mechanistic insight that may help identify individuals who are sensitive to environmental exposures [19, 20]. BPD is a complex disorder, and because the contribution of each gene in a complex trait may be relatively minor, identification of each of the genes that ultimately contribute to a complex trait is a major challenge [21]. Furthermore, susceptibility genes interact with multiple environmental exposures or stimuli related to the etiology of a disease. In order to better define the genetic contribution to BPD susceptibility, we have chosen gene candidates a priori that have biological plausibility to contribute to the pathogenesis of BPD. These phenotypes can be tested using in vivo/in vitro in model systems and in the Buenos Aires D-BPD population. We have also performed a genome-wide association study (GWAS) of hyperoxia-induced acute lung injury in neonatal inbred mice which recapitulates some characteristics of BPD. This gene discovery approach identified a number of novel genes that have been tested and confirmed to have a role in susceptibility to acute lung injury in neonatal mice [22]. The combination of gene discovery and biologically plausible genes provides a panel of candidates that may be used to screen VLBW infants and, potentially, develop more precise intervention/prevention strategies in the treatment of BPD. Lastly, evaluating ancestry indicative markers is an excellent way to discover novel genes underlying complex diseases [23] like premature lung disease, and the availability of infant-parent triad will allow us to pursue those investigations.

### Analytic approach by machine learning

A central problem regarding the phenotypic characterization of BPD relates to the current definition of the disease: oxygen requirement [24]. This operational definition fails to convey the diverse underlying pulmonary pathologies, the varying degrees of pathology between individual preterm infants due to differences in pulmonary development, the presence of lung fibrosis (and resulting changes in lung compliance), the severity of lung vascular remodeling (and resulting pulmonary hypertension) and the degree of tracheomalacia and/or bronchomalacia. These factors may vary widely between individual infants and perhaps even in the same infant over time given that BPD is a multifactorial disorder superimposed upon the developing lung. These realities suggest that BPD is most likely to be a superficial umbrella term that encompasses related but different conditions caused by distinct underlying pathophysiological mechanisms. The large amounts of data that will be amassed during the present study and the urgent need for more stringent dissection of the causes and outcomes under the BPD diagnosis supports the use of machine learning [25] for assessing these possible sub variants (endotypes). These endotypes will be generated employing latent class analysis (LCA) [26, 27], a data-driven, hypothesis-generating approach. *Clusters (endotypes) will be constructed employing longitudinal data without any* a priori *classification such as the canonical labels “severe” or “mild” BPD.* To this end, patient-specific data will be used for the construction of trajectories. Each trajectory will be based upon the time course of the assessed variables including the degree of respiratory support, growth, infection, early childhood respiratory function and symptoms. The dimensionality of these variables will be reduced using principal component analysis [28]. The use of LCA guarantees the acquisition of unbiased endotypes enabling circumvention of simple clinical phenotypic characterization based upon a single dimension of the disease. Thus, the resulting endotypes will encompass all relevant descriptors of disease progression. *Once the endotypes, or clusters, are generated, the next step will be the segregation of transversal (non time-dependent) variables among the different clusters including, but not limited to,* genetic markers, environmental conditions, sex, chorioamnionitis and other pathophysiological outcomes. *These transversal variables should allow a better understanding of the molecular basis underlying individual endotypes. These data could lead to better diagnostics and the eventual possibility of developing personalized treatments for each endotype.* Thus, machine learning is one of the novel fundamental approaches of the D-BPD program that will enable the team to propose new definitions that will be used in clinical study design, drug development and assessments of novel therapies as part of a personalized medicine therapeutic approach for each individual patient.

### Molecular basis of disease onset and severity

One of the main objectives of our machine learning approach is to characterize the underlying endotypes in infants with a diagnosis of BPD. Bridging the gap between endotypes and causal mechanisms is a major challenge [29]. We will tackle this issue by utilizing identified candidate genes for disease. The connection between endotypes and candidate genes will be assessed, enabling the achievement of the ultimate goal of the D-BPD program: to define the molecular basis that contribute to endotypes of BPD. This knowledge will facilitate the pursuit of specific treatments, ranging from improved palliative care to the development of long-term projects for target-specific drug design. To this end, the identified variants/mutants will be classified using bioinformatics [30]. The first step consists of assessing the effects of genetic mutations on gene expression at the level of transcription, splicing or mRNA half-life, and protein structure/function [30–32]. Candidate proteins will be studied by employing a combined in silico/in vitro approach. The effect of the mutations will be evaluated on the basis of previous reports regarding functional data, interaction analysis with other proteins or RNA/DNA, and available data from system biology or structural data when NMR and/or crystal structures of the candidate proteins are available. Bioinformatics, homology modelling and molecular dynamic simulations will be applied in parallel with in vitro approaches that consist of recombinant expression and purification of candidate proteins and/or individual subdomains. The wild-type and relevant mutants will be assessed at the structure-dynamics-function level and will encompass a complete battery of spectroscopic and biophysical characterization methods including far-UV circular dichroism spectroscopy, vibrational spectroscopy, fluorescence and spectroscopy in order to determine structure and stability. For each specific protein, depending on their known functions, individual protocols for assessment of function of the mutant proteins will be designed including, but not limited to, interaction assays for complex formation, redox properties and enzymatic functions.

### Study approval and oversight

The multi-center D-BPD protocol and consent, additional information to be completed by the participants, such as survey instruments or questionnaires, proposals, and any other advertising/contracting material has been be submitted to the NIEHS IRB and all participating local IRBs for approval in writing. The protocols, consent, and survey instruments are reviewed annually for progress and compliance. We will submit and obtain approval from the NIEHS IRB and all participating local IRBs for all subsequent modifications to the protocol, informed consent documents, and any documentation pertaining to the study. We are responsible for obtaining approval from the NIEHS IRB and all participating local IRBs of the ongoing continuing review throughout the entire duration of the study. We will notify the NIEHS IRB and all participating local IRBs of serious adverse events and protocol violations per their requirements.

### Training and quality control

Since the inception of the study, Fundacion INFANT has held bi-weekly training webinars with the research team from each site to ensure uniform approaches to data and specimen collection.

### Summary and progress through enrollment

**Fig. 4:**
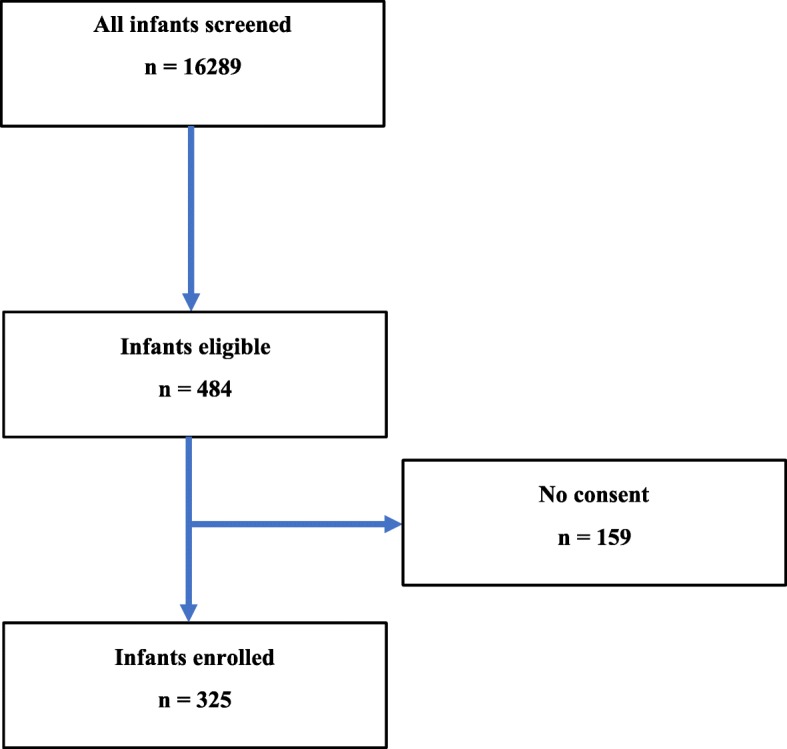
D-BPD cohort diagram

Enrollment began in June, 2013 and is ongoing **(**Fig. 4**)**. Consent rates have ranged from 45 to 90% by center (67% for the overall consortium) for a total enrollment of 325 participants. The biospecimen archive of DNA, cord blood, physiologic testing results, and breadth of the investigative teams has prompted the initiation of several ancillary studies that have added dimensions to the original D-BPD design (Table 3).

**Table 3:** Ancillary projects arising from D-BPD

Project	
Early prediction of asthma inception by FOT	
Lung function trajectories of VLBW infants	
Mitochondrial dysfunction and impact in BPD development	
Micronutrients status and effect on long term respiratory outcomes	
Mitochondria DNA variants and susceptibility for lung disease	
Forced oscillatory testing and prediction of asthma in BPD	

## Discussion

In summary, the current gap in understanding BPD as a complex multi-trait spectrum of different disease endotypes will be addressed by a bedside-to-bench and bench-to-bedside approach in the D-BPD program. Other observational programs have been successful in identifying perinatal and clinical risk factors and have very elegantly described respiratory physiology in infants [6, 33]. A few important assets that distinguish our program from others include: 1) The recruitment of case/parent triads which makes it possible to perform transmission/disequilibrium tests to identify preferential transmission of alleles from parent to affected child within different triads (comprising an affected child plus two parents). The transmission/disequilibrium test (TDT) considers parents who are heterozygous for an allele associated with disease and evaluates the frequency with which that allele or its alternate is transmitted to affected offspring [34]. Compared with conventional tests for linkage, the TDT has the advantage that it does not require data either on multiple affected family members or on unaffected sibs. Moreover, the use of parental data, instead of nonrelated controls avoids ethnic confounding, even if the parents represent a mixture of ethnic backgrounds. 2) We plan to study lung function utilizing standard spirometry testing and novel lung function evaluations with Forced Oscillatory Testing beyond the first year of life. Therefore, our studies can extend the characterization of lung development into childhood and, consequently, identify manifestations of premature lung disease that may not be apparent until later in life. 3) Finally, the ultimate goal of the endotype discovery paradigm is to build upon the foundations earlier studies, including PROP, to identify novel pathways that contribute to pulmonary outcomes in prematurely born infants. Specifically, we will develop machine learning algorithms to identify endotypes from our cohort to enable the use of an unbiased, hypothesis generating approach. Similar approaches have recently been used to uncover disease endotypes “hidden” under the same umbrella term (e.g.: fever or asthma) [16]. Our hypothesis is that this will disaggregate premature lung disease into several subgroups with different etiologies and prognoses hidden under the BPD definition to date. A limitation of our program is a lack of standardized physiologic testing during the NICU course including a room air challenge at 36 weeks PCA. The room air challenge enables identification of infants with immature control of breathing and/or a weak chest wall/airway. Given the longitudinal nature of our study and the development of trajectories for clustering, we are confident that analysis of the longitudinal data will enable the unbiased identification of the above-referenced infants.

Overall the D-BPD Program will provide enhanced understanding of mechanisms, evolution and consequences of lung diseases in preterm infants. The D-BPD program represents a unique opportunity to combine the expertise of biologists, neonatologists, pulmonologists, geneticists and biostatisticians to examine the disease process from multiple perspectives with a singular goal of improving outcomes of premature infants.

## Additional file

Additional file 1: Investigators and Research Staff. (DOCX 12 kb)

## Data Availability

The datasets used and/or analyzed during the current study will be available from the corresponding author on reasonable request.

## Abbreviations

BPD: Bronchopulmonary dysplasia
D-BPD: Discovery bronchopulmonary dysplasia
FOT: Forced oscillatory test
NICU: Neonatal intensive care unit
NIEHS: National Institute of Environmental Sciences
PCA: Post-conceptional age
ROP: Retinopathy of prematurity
VLBW: Very low birth weight
