# Supplementary material for: The discovery BPD (D-BPD) program: study protocol of a prospective translational multicenter collaborative study to investigate determinants of chronic lung disease in very low birth weight infants
Source: BMC Pediatr. 2019 Jul 6;19:227. doi: 10.1186/s12887-019-1610-8 (PMC6612113; doi:10.1186/s12887-019-1610-8)
Supplement: Supplementary file 1 — Investigators and Research Staff. (DOCX 12 kb) [file 12887_2019_1610_MOESM1_ESM.docx]

**Additional file 1**

**Investigators and Research Staff**

University of Alabama at Birmingham (UAB)

***Investigators and research staff***

Trent E. Tipple, MD

Gaston Ofman, MD

Fundacion INFANT

***Investigators and research staff***

Mauricio T. Caballero, MD

Damian Alvarez Paggi, Ph.D.

Fernando P. Polack, MD

Alejandra Bianchi

Florencia Nowogrodski, MS

NIEHS

***Investigators and Research Staff***

Douglas A. Bell, Ph.D.

Pierre Bushel, Ph.D.

Steven R. Kleeberger, Ph.D.

Jianying Li, Ph.D.

Jacqueline Marzec, M.S.

Jennifer Nichols, Ph.D.

Min Shi, Ph.D.

Kirsten Verhein, Ph.D.

Xuting Wang, Ph.D.

Clarice Weinberg, Ph.D.

FUNDASAMIN

Luis M. Prudent, MD

Nestor Vain, MD

Pontificia Universidade Católica do Rio Grande do Sul

Marcus H. Jones, MD

D-BPD Program Network

Gonzalo Mariani, MD

Mariana Sorgetti, MD

Jorge Digregorio, MD

Elba Lopez Turconi, MD

Cristina Osio, MD

Fernanda Galletti, MD

Mariangeles Quiros, MD

Andrea Brum, MD

Santiago Lopez Garcia, MD

Silvia Garcia, MD
